# Anti-Inflammatory Effect of Pestalotic Acid A Derived from *Pestalotiopsis vismiae,* an Endophytic Fungus of *Ilex prenatal*, in Lipopolysaccharide-Stimulated RAW264.7 Cells

**DOI:** 10.3390/biomedicines13061445

**Published:** 2025-06-12

**Authors:** Da Young Hwang, Dae-Won Ki, Dae-Cheol Choi, Bong-Sik Yun, Yoon Hee Kim

**Affiliations:** 1 Department of Food and Nutrition, College of Engineering, Daegu University, Gyeongsan-si 38453, Republic of Korea; suga8037@naver.com; 2 Division of Biotechnology, Jeonbuk National University, Iksan-si 54596, Republic of Korea; dwki0916@jbnu.ac.kr (D.-W.K.); ell612@naver.com (D.-C.C.)

**Keywords:** endophytic fungi, inflammation, macrophage, pestalotic acid A, Japanese holly

## Abstract

**Background/Objectives:** Pestalotic acid A (PAA), a polyketide derived from *Pestalotiopsis vismiae*, an endophyte of the Japanese holly (*Ilex crenata*), is known to exhibit known antimicrobial activity, but its anti-inflammatory properties remain uncharacterized. This study aimed to investigate the anti-inflammatory effects of PAA in lipopolysaccharide (LPS)-stimulated murine macrophages, RAW264.7 cells. **Methods:** PAA was isolated from *P. vismiae* endophytes of *Ilex crenata,* and its structure was confirmed. RAW264.7 macrophages were treated with 0–50 μM of PAA in the presence of 100 ng/mL LPS. Cell viability was assessed by MTS assay; nitric oxide (NO) production was measured via Griess reagent; interleukin (IL)-6, IL-1β, and tumor necrosis factor (TNF) were quantified by enzyme-linked immunosorbent assay. Protein expression of inducible NO synthase (iNOS), nuclear factor (NF)-κB p65 phosphorylation, and related signaling proteins was evaluated by Western blot analysis and immunofluorescence staining. **Results:** PAA significantly increased macrophage viability and dose-dependently inhibited the release of NO by alleviating the protein expression of iNOS in LPS-treated RAW264.7 cells. Furthermore, PAA suppressed the release of IL-6, IL-1β, and TNF induced by LPS. Western blot and immunofluorescence results also indicated that PAA blocked the p65 subunit phosphorylation of NF-κB, which is one of the underlying mechanisms of the anti-inflammatory action of pestalotic acid A. **Conclusions:** PAA exerts potent anti-inflammatory effects in LPS-stimulated macrophages via inhibition of the NF-κB pathway, highlighting its potential as a natural therapeutic agent for inflammatory diseases.

## 1. Introduction

Endophytic fungi are organisms that live symbiotically within plant tissues, such as those of leaves, stems, and roots, without harming the host plant. These fungi exhibit high biological diversity and contribute to plant growth by enhancing nutrient uptake and improving stress tolerance [1]. They produce a range of secondary metabolites, including steroids, flavonoids, and alkaloids, which enhance plants’ resistance to pathogens and herbivores, thereby promoting overall plant health [2,3].

These fungi are promising sources of novel antimicrobial agents, with compounds like prenylated indole alkaloids showing efficacy against multidrug-resistant pathogens [4]. Common endophytes include *Pestalotiopsis*, *Fusarium*, *Aspergillus,* and *Penicillium*, which co-evolve with their hosts in unique environments and produce various bioactive substances [5].

Endophytic fungi represent substantial biological resources capable of producing diverse bioactive compounds. Their potential applications span multiple fields, including drug discovery. Therefore, the isolation and identification of functional compounds from endophytic fungi are of crucial importance in natural product chemistry and pharmaceutical sciences [6].

*Ilex crenata*, commonly known as Japanese holly, is a species of holly characterized by small, evergreen leaves and a dense growth habit. It is frequently used in landscaping and ornamental gardening owing to its aesthetic appeal and adaptability [7]. Endophytic fungi associated with *Ilex crenata* can produce unique metabolites, including triterpenoids, which have potential health benefits [8]. The metabolites produced by these endophytic fungi may exhibit antimicrobial activities that contribute to the plant’s defense mechanisms [9].

This study aimed to isolate secondary metabolite from *Pestalotiopsis vismiae (P. vismiae),* an endophytic fungus associated with *Ilex crenata,* and to investigate the effects of the isolated secondary metabolite on inflammatory response in lipopolysaccharide (LPS)-stimulated murine macrophages, along with their underlying mechanisms. Pestalotic acid A (PAA) is a known polyketide-type secondary metabolite that has previously been isolated from *Pestalotiopsis* species. In this study, it was identified as the major constituent of *P*. *vismiae* extract, marking the first report of its isolation from this fungal strain. Although PAA has been evaluated for antimicrobial activity in previous studies, it has not been shown to exhibit significant efficacy [10]. In preliminary experiments, we measured the effect of four polyketide-types secondary metabolite isolated from *P*. *vismiae* extract on production of nitric oxide (NO) and macrophage cell viability. Among the four polyketide-types, only PAA exhibited an inhibitory effect on production of NO without cell cytotoxicity (Figures S1 and S2). Herein, we report for the first time the anti-inflammatory activity of PAA, based on its effects in LPS-stimulated murine macrophages and molecular mechanism, suggesting its potential relevance in inflammation-related conditions.

## 2. Materials and Methods

### 2.1. Materials

LPS derived from *Salmonella abortusequi* was purchased from Sigma-Aldrich (St. Louis, MO, USA). The Dulbecco’s modified Eagle’s medium (DMEM), fetal bovine serum (FBS), penicillin, and streptomycin used in this study were obtained from Hyclone (Logan, UT, USA). The Bio-Rad Protein Assay kit was acquired from Bio-Rad Laboratories, Inc. (Hercues, CA, USA). The antibodies used in this study were anti-nuclear factor (NF)-κB p65 (D14E12) rabbit monoclonal, anti-phospho-NF-κB p65 (Ser536) (93H1), anti-IκBα (L35A5), anti-phospho- IκBα (Ser32/36) (5A5), anti-p38 MAPK (mitogen-activated protein kinase) (D13E1), anti- phospho-p38 MAPK (Thr180/Tyr182), anti-SAPK/JNK, anti-phospho-SAPK/JNK (Thr183/Tyr185) (G9), anti-p44/42 MAPK (Erk1/2), anti-phospho-p44/p42 MAPK (Erk1/2) (Thr202/Tyr204), anti-PI3 kinase p85 (19H8), anti-phospho-PI3 kinase p85 (Tyr458)/p55 (Tyr199) (E3U1H), anti-AKT, anti-phospho-AKT (Ser473), inducible nitric oxide synthase (iNOS) (D6B6S) rabbit mAB (#13120), Lamin A/C (4C11) (Cell Signaling Technology, Danvers, MA, USA), and anti-β-actin mouse monoclonal (Proteintech, Wuhan, China).

### 2.2. Source of Endophytic Fungi

Leaves and small branches of *Ilex crenata* were collected from healthy plants in plantation fields and forested areas at the Jeonbuk National University specialized campus, Jeonbuk province, Republic of Korea, in November 2021. Plant specimens were authenticated and preserved as voucher specimens at the Jeonbuk National University Herbarium.

### 2.3. Isolation and Identification of Endophytic Fungi

The collected plant materials were transported to the Natural Products Chemistry Laboratory at Jeonbuk National University, where they were washed with a mild detergent and running tap water, air-dried, and cut into small fragments using sterile surgical blades. The fragments were surface-sterilized by sequential immersion in 95% ethanol for 30 s, followed by 5% sodium hypochlorite for 5 min, another 30 s in 95% ethanol, and rinsing with sterile distilled water for 3–5 s. Surface-sterilized fragments were then plated onto cornmeal agar supplemented with 50 mg/L tetracycline and ampicillin to inhibit bacterial growth, then incubated at 25 °C. Hyphal tips from the plant fragments were transferred to potato dextrose agar (PDA) for purification using the hyphal tip method, resulting in 50 isolated endophytic fungi.

One isolate was selected for molecular identification. Genomic DNA was extracted, and the internal transcribed spacer (ITS) regions of the ribosomal RNA gene were amplified and sequenced by Macrogen Inc., Seoul, Republic of Korea. The resulting ITS sequences were compared with those in the GenBank database using the BLAST search tool (NCBI BLASTN, version 2.6.0+). The ITS5 region (1441 bp) showed 100% sequence identity with the *P. vismiae* strain PN3CW (GenBank accession number EF055221.1), whereas the ITS4 region (1775 bp) exhibited 99% identity with the *Pestalotiopsis* sp. isolate JSM 06261592 (GenBank accession number KY086253.1). These high levels of sequence similarity confirmed the identification of the isolate as *P*. *vismiae*.

### 2.4. Cultivation and Extraction of Pestalotiopsis Vismiae

The fungal strain *P. vismiae* was cultured on a PDA medium for a week at 27 °C. It was then inoculated into a 25 L flask and cultured at 27 °C for 3 weeks with shaking at 120 rpm. The culture broth of *P*. *vismiae* was filtered to separate the broth filtrate from the mycelium. The broth filtrate was extracted with ethyl acetate (EtOAc), and the mycelium was extracted with acetone for 24 h at room temperature. The acetone extract was filtered and evaporated under reduced pressure, after which the residue was partitioned between EtOAc and water (H_2_O).

### 2.5. Isolation and Purification of PAA

The EtOAc soluble layer (5.78 g) was subjected to silica gel column chromatography and eluted with chloroform–methanol (CHCl_3_:MeOH [50:1, 20:1, 10:1, 5:1, and 2:1, *v*/*v*, stepwise]). Preliminary thin-layer and high-performance liquid chromatography analyses identified a major fraction, designated as fraction A (1.41 g). This fraction was subjected to reversed-phase medium-pressure liquid chromatography (MPLC) on a Combiflash RF system (Teledyne Isco, Lincoln, NE, USA), using a RediSep^®^ Rf C_18_ column (130 g), followed by gradient elution with aqueous methanol under the following conditions: 0–10 min, 0% methanol; 10–15 min, 0–30% methanol; 15–65 min, 30–70% methanol; 65–70 min, 70–100% methanol; and 70–80 min, 100% methanol, at a flow rate of 15 mL/min with monitoring using a UV detector at 254 nm to yield sub-fraction *F*_A1_. Sub-fraction *F*_A1_ was further subjected to Sephadex LH-20 column chromatography and eluted with MeOH to yield sub-fraction *F*_A1-1_. Sub-fraction *F*_A1-1_ was further purified by Sephadex LH-20 column chromatography and eluted with MeOH to yield sub-fraction *F*_A1-1_. This fraction was then subjected to reversed-phase medium-pressure liquid chromatography (MPLC) using a RediSep^®^ Rf C_18_ column (86 g), followed by isocratic elution with 50% aqueous MeOH at a flow rate of 5 mL/min and monitoring with a UV detector at 254 nm to afford PAA (101.1 mg).

### 2.6. LC/ESI-MS Analysis of PAA

ESI-MS analysis was performed using an Agilent Technologies 6410 Triple Quadrupole LC/MS system equipped with an electrospray ionization (ESI) source operating in negative ion mode. The separation was carried out on a Cosmosil RP-18 column (4.6 × 150 mm) with gradient elution of MeOH/H_2_O (20%, 0–2 min), 20–80% MeOH (2–17 min), 80–20% MeOH (17–18 min), and 20% MeOH (18–20 min). The flow rate was set to 1.0 mL/min, and UV detection was performed at 254 nm. The ESI voltage was set at +3.0 kV. Under these conditions, PAA was detected at a retention time of 15.7 min, showing pseudo-molecular ion peaks at *m/z* 349.3 [M–H]^–^ and 699.3 [2M–H]^–^.

### 2.7. Analytical HPLC for Purity Determination

The purity of PAA (1.0 mg) was assessed by reverse-phase HPLC using a TSKgel ODS column (4.6 mm i.d. × 150 mm, Tosoh, Tokyo, Japan), a pump (L-2130, Hitachi High-Tech, Tokyo, Japan), and a photodiode array detector (L-2455, Hitachi High-Tech, Tokyo, Japan). The elution was performed using a gradient solvent system of 10% to 100% methanol containing 0.04% trifluoroacetic acid at a flow rate of 1.0 mL/min. PAA was detected at a retention time of 20.9 min with a purity of 97.2%, as calculated based on peak area. The HPLC chromatogram is provided in the Supplementary Information (Figure S9).

### 2.8. Cells and Cell Culture

RAW264.7 cells, murine macrophage cells, were obtained from the Korean Cell Bank (Seoul, Republic of Korea). RAW264.7 cells were grown in DMEM supplemented with 10% FBS, 100 U/mL penicillin, and 100 mg/mL streptomycin. Cells were maintained at 37 °C in a humidified incubator under 5% CO_2_. The vehicle (LPS-untreated group) and the 0 μM PAA (only 100 ng/mL LPS-treated group) were also treated with equal amounts of dimethyl sulfoxide (DMSO), with maximum DMSO concentration maintained at <0.001% *v*/*v*.

### 2.9. Cell Proliferation Assay

The effect of PAA on cell proliferation was assessed using CellTiter96^®^ Aqueous One Solution Assay of cell proliferation (Promega, Madison, WI, USA). RAW264.7 cells were plated at a density of 1 × 10^4^ cells per 200 μL in a 96-well plate, and PAA was added to each well at concentrations ranging from vehicle to 50 μM in DMEM containing 1% FBS. After 24 h incubation, cell proliferation was measured according to the manufacturer’s instructions at 490 nm.

### 2.10. Measurement of Inflammatory Mediators

The amounts of representative inflammatory mediators (NO, interleukin [IL]-6, IL-1β, tumor necrosis factor-α [TNF]) released by the mouse macrophages were determined by measuring the concentrations of each substance in the cell culture supernatant. RAW264.7 cells were plated at a density of 1 × 10^5^ cells per 1 mL per well and incubated overnight. They were then treated with 0–50 μM of PAA in the presence of 100 ng/mL LPS and incubated for another 24 h. The culture supernatant was collected and assayed using the Griess reagent system (Promega), mouse IL-6 enzyme-linked immunosorbent assay (ELISA) kit, mouse TNF-α ELISA kit (eBiosciences, San Diego, CA, USA), and Quantikine^TM^ ELISA Mouse IL-1β/IL-1F2 Immunoassay (R&D Systems, Minneapolis, MN, USA), according to the manufacturer’s instructions.

### 2.11. Preparation of Whole-Cell Lysates

RAW264.7 cells were plated at a density of 1 × 10^5^ cells per 1 mL per well and incubated overnight. They were then treated with 0–50 μM of PAA in the presence of 100 ng/mL LPS and incubated for either 4 or 24 h. For whole-cell lysate preparation, cells were lysed in a lysis buffer containing 50 mM Tris-hydrochloric acid (pH 7.5), 150 mM sodium chloride, 1% Triton X-100, 1 mM ethylenediamine tetra-acetic acid, 50 mM sodium fluoride, 30 mM sodium pyrophosphate, 1 mM phenylmethanesulfonyl fluoride, 2 mg/mL aprotinin, and 1 mM pervanadate. The lysates were clarified by centrifugation at 12,000× *g* for 15 min at 4 °C and subsequently stored at –20 °C until further use.

### 2.12. Western Blotting Analysis

Whole-cell lysates (10 or 15 μg protein per lane) were separated by 10% sodium dodecyl sulfate-polyacrylamide gel electrophoresis and transferred to Immobilon^®^ transfer membranes (0.45 μm, Millipore, Billerica, MA, USA). After blocking with 1X blocking buffer (Biofact Biofactory, Daejeon, Republic of Korea) for 1 h, the membranes were incubated with the primary antibody at 4 °C overnight. The membranes were then washed with Tris-buffered saline–Tween 20 solution and incubated for 1 h with anti-rabbit immunoglobulin G (IgG) (#7074S), mouse anti-rabbit IgG (Conformation Specific) (L27A9), or anti-mouse IgG conjugated with horseradish peroxidase. After washing, the protein bands were visualized using an enhanced chemiluminescence reagent (Thermo Fisher Scientific, Rockford, IL, USA) and Fusion Solo S (Vilber Smart Imaging, Marne-la-Vallée, France). Band intensity was assessed using ImageJ software Version 1.53k (NIH, Bethesda, MA, USA).

### 2.13. Immunofluorescence Staining

RAW264.7 cells were plated onto glass coverslips pretreated with tissue cultures at a density of 1 × 10^4^ cells per 200 μL per well, then incubated overnight. They were then treated with 0–50 μM of PAA in the presence of 100 ng/mL LPS and incubated for an additional 24 h. Treated cells were fixed with 4% formaldehyde, lysed with 0.3% Triton^TM^ X-100, and incubated with a primary antibody against phosphor-NF-κB p65 (Ser536) (93H1) (Cell Signaling Technology), followed by incubation with anti-rabbit IgG (H + L), F(ab’)_2_ Fragment (Alexa Fluor^®^ 488 Conjugate) (Cell Signaling Technology). Nuclei were stained with ProLong Gold Antifade Reagent 4′,6-diamidino-2-phenylindole (DAPI) (Cell Signaling Technology). Finally, a Microscope Cover Glass (Marienfeld, Germany) was placed on a frosted slide glass, and samples were examined with an optical microscope (Leica DM2700 M, Leica, Wetzlar, Germany).

### 2.14. Statistical Analysis

Statistical analysis was performed using GraphPad Prism Version 5.0 software (GraphPad, San Diego, CA, USA). Data were presented as the mean ± standard error of the mean. Differences between mean values of multiple groups were analyzed using a one-way analysis of variance followed by Dunnett’s test. Statistical significance was established at *p* < 0.05.

## 3. Results

### 3.1. Identification of Active Compound

The active compound was isolated from the EtOAc soluble portion, which was obtained as a brown oil. Negative electrospray ionization mass spectrometry analysis demonstrated pseudo-molecular ion peaks at *m/z* 349.3 [M−H]^−^ and 699.3 [2M−H]^−^ (Figure S3). The proton nuclear magnetic resonance (^1^H NMR) spectrum (Figure S4) active compound exhibited three olefinic proton signals at *δ*_H_ 6.83 (td, *J* = 7.5, 1.3 Hz, H-3), 6.66 (dt, *J* = 15.9, 1.2 Hz, H-11), and 6.54 (dt, *J* = 15.9, 7.0 Hz, H-12). Additional signals included oxygenated methine and methylene protons at *δ*_H_ 4.85 (s, H-6), 4.48 (d, *J* = 11.7 Hz, H-19), 4.28 (d, *J* = 11.7 Hz, H-19), and 3.35 (s, H-10); five methylene proton signals at *δ*_H_ 3.15 (dd, *J* = 15.8, 7.6 Hz, H-4), 2.72 (dd, *J* = 15.8, 7.6 Hz, H-4), 2.29 (dd, *J* = 15.0, 8.0 Hz, H-13), 1.52 (m, H-14), and 1.35 (m, H-15 and H-16); and two methyl proton signals at *δ*_H_ 1.89 (s, H-18) and 0.92 (t, *J* = 7.0 Hz, H-17) (Table S1).

The ^13^C NMR (Figure S5) and HMQC (Figure S6) spectra of the active compound displayed signals corresponding to two carbonyl carbons at *δ*_C_ 197.0 (C-9) and 171.2 (C-1); six olefin carbons at *δ*_C_ 151.2 (C-7), 143.1 (C-12), 135.9 (C-3), 132.8 (C-2), 130.2 (C-8), and 127.1 (C-11); one oxygenated quaternary carbon at *δ*_C_ 64.5 (C-5); two oxygenated methine carbons at *δ*_C_ 66.3 (C-6) and 59.0 (C-10); one oxygenated methylene carbon at *δ*_C_ 55.0 (C-19); five methylene carbons at *δ*_C_ 35.0 (C-13), 32.6 (C-15), 30.9 (C-4), 29.6 (C-14), and 23.7 (C-16); and two methyl carbons at *δ*_C_ 14.4 (C-17) and 12.9 (C-18) (Table S1).

**Figure 1:**
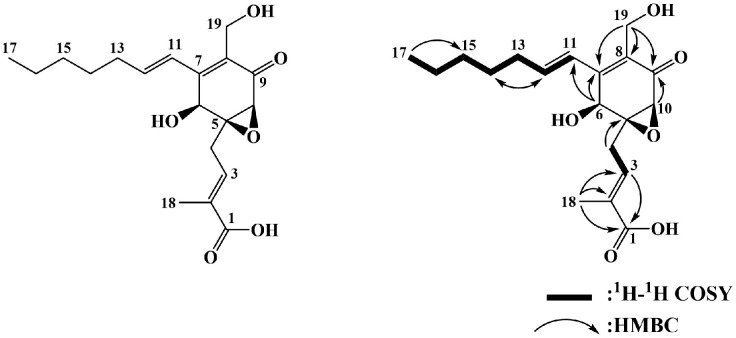
Chemical structure and two-dimensional NMR correlations of pestalotic acid A.

The ^1^H and ^13^C NMR data of the active compound were consistent with those of pestalotic acid A (PAA). These spectral data closely matched previously published findings [11]. The chemical structure of PAA was confirmed through the interpretation of two-dimensional NMR spectra, including ^1^H–^1^H correlation spectroscopy and heteronuclear multiple-bond correlation spectroscopy (Figure 1 and Figures S7 and S8). The purity of PAA was confirmed to be 97.2% by analytical HPLC analysis, supporting the reliability of the structural assignment (Figure S7).

### 3.2. Pestalotic Acid A Increases Macrophage Viability

**Figure 2:**
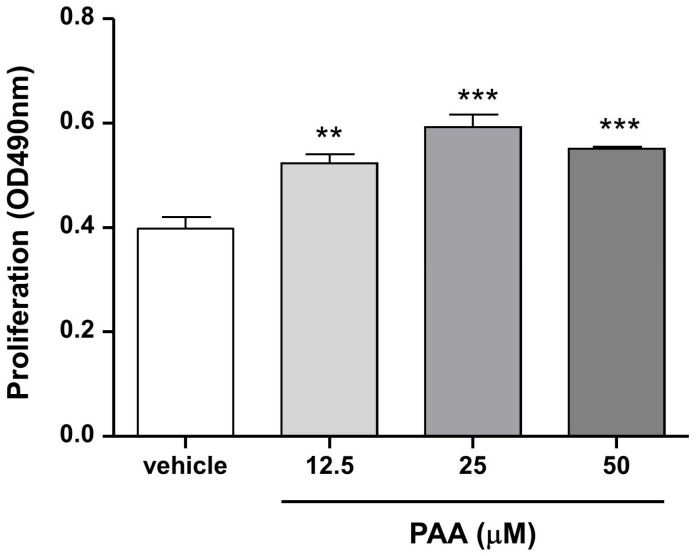
Pestalotic acid A increases proliferation of RAW264.7 cells, a murine macrophage cell line. Results are reported in the form of mean ± standard error of the mean for three independent experiments (*n* = 4). Statistical significance was determined based on differences when compared with vehicle (** *p* < 0.01, *** *p* < 0.001).

Macrophages play an important role in inflammatory response [12,13]. To investigate the effects of PAA on inflammation, murine macrophages (RAW264.7 cells) were used. Initially, the effect of PAA on cell proliferation in RAW264.7 cells was examined (Figure 2). The absorbance intensities at vehicle and 50 μM PAA were 0.40 ± 0.02 and 0.55 ± 0.01 (*** *p* < 0.001), respectively. These results indicated that PAA significantly increased macrophage viability. A concentration range of 0–50 μM was used in subsequent experiments.

### 3.3. Pestalotic Acid A Inhibits Release of Nitric Oxide (NO) by Suppressing iNOS Expression in LPS-Stimulated RAW264.7 Cells

**Figure 3:**
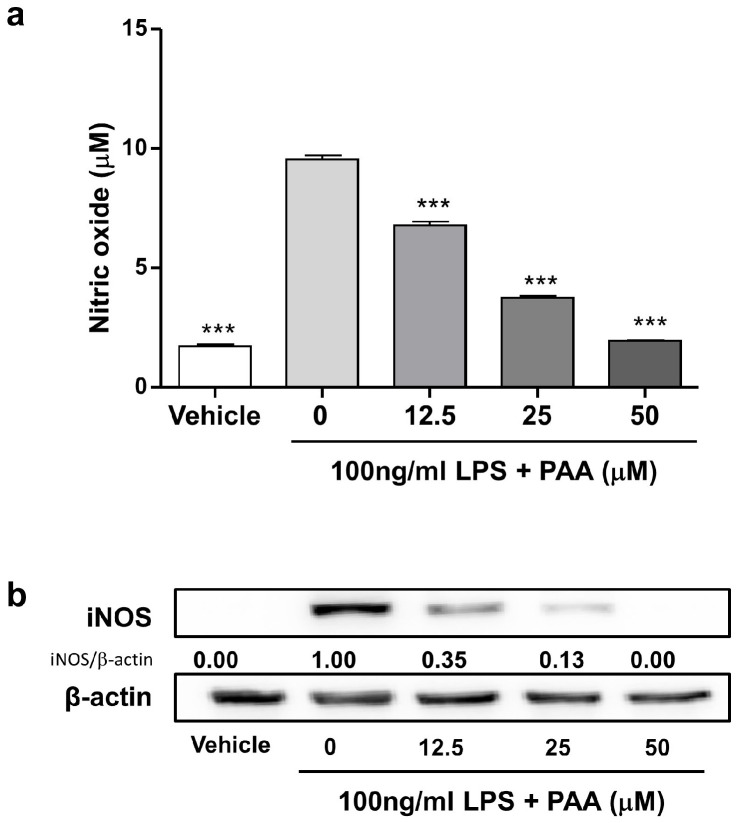
Pestalotic acid A inhibits production of NO by suppressing its synthesizing enzyme in LPS-stimulated RAW264.7 cells. In the cell culture supernatant, the levels of NO (**a**) were determined by methods described in the Materials and Methods section (*n* = 3). (**b**) In each whole-cell lysate, 10 μg proteins were subjected to 10% SDS-PAGE for iNOS expression. β-actin expression served as a loading control. The bands were quantified using image analysis software, and their relative intensity was expressed as iNOS/β-actin (*n* = 1). Statistical significance was demonstrated based on differences when compared with 0 μM pestalotic acid A (*** *p* < 0.001).

To assess the effect of PAA on LPS-induced inflammatory response in RAW264.7 cells, the cells were exposed to 100 ng/mL LPS in the presence of PAA, and the levels of released nitrite, a stable metabolite of NO, were measured in the cell culture supernatant. As shown in Figure 3a, PAA inhibited LPS-induced NO production starting from a concentration of 12.5 μM in a dose-dependent manner (0 μM; 9.53 ± 0.18 mM, 12.5 μM PAA; 6.77 ± 0.18 mM, 25 μM PAA; 3.74 ± 0.09 mM, 50 μM PAA; 1.94 ± 0.04 mM) (*** *p* < 0.01).

To elucidate the mechanism by which PAA suppresses LPS-induced NO production, its effect on iNOS protein expression was evaluated in whole-cell lysate using Western blot analysis. As presented in Figure 3b, LPS treatment resulted in a marked increase in iNOS protein expression in RAW264.7 cells (iNOS/β -actin intensity: 1.00). PAA treatment inhibited LPS-induced iNOS protein expression at 12.5 μM (iNOS/β-actin intensity: 0.35), 25 μM (iNOS/β-actin intensity: 0.13), and 50 μM (iNOS/β-actin intensity: 0.00) concentrations of PAA, respectively. These findings support the conclusion that PAA inhibits the release of NO by suppressing the expression of its synthesizing enzyme in LPS-stimulated RAW264.7 cells.

### 3.4. Pestalotic Acid A Inhibits Pro-Inflammatory Cytokine Production in LPS-Stimulated RAW264.7 Cells

**Figure 4:**
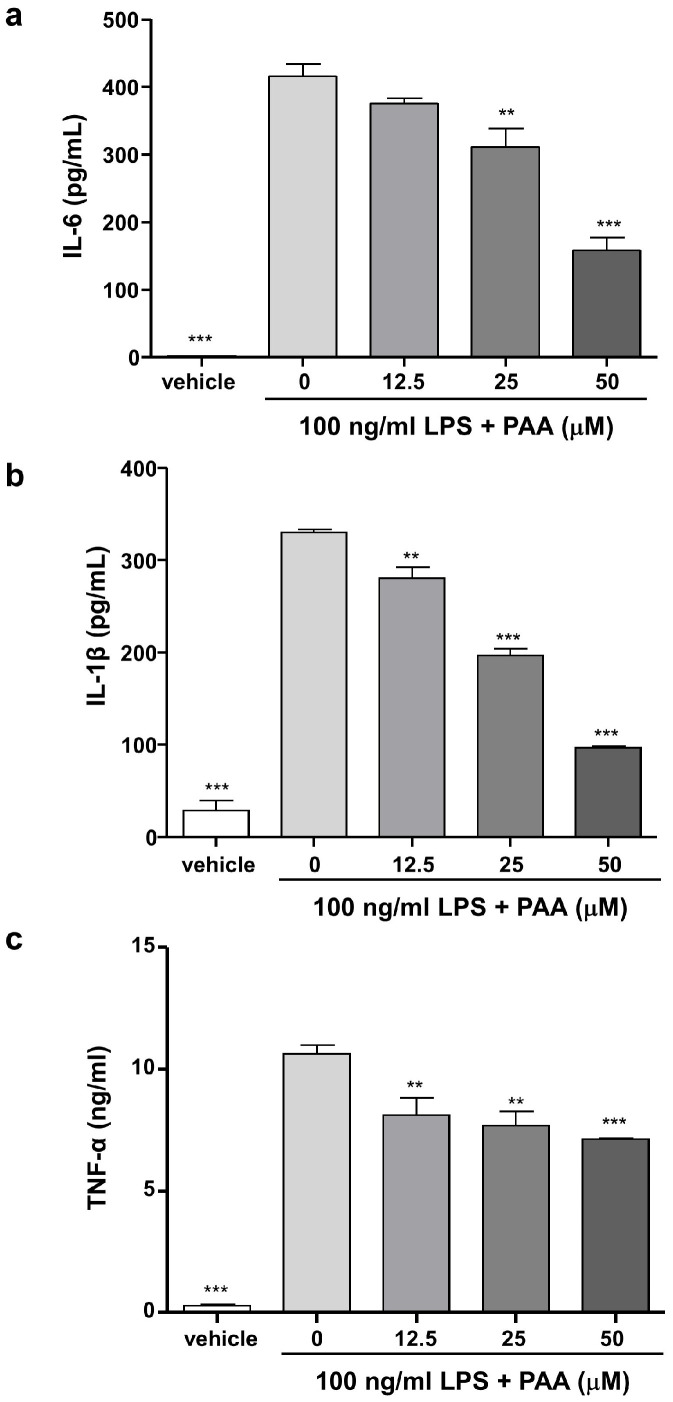
Pestalotic acid A suppresses production of inflammatory cytokines in LPS-stimulated RAW264.7 cells. In the cell culture supernatant, IL-6 (**a**), IL-1β (**b**), and TNF (**c**) were determined by methods described in the Materials and Methods section (*n* = 3). Statistical significance was determined based on differences when compared with 0 μM PAA (** *p* < 0.01, *** *p* < 0.001).

Subsequently, the effects of PAA on the production of the pro-inflammatory cytokines IL-6, IL-1β, and TNF were investigated. The concentrations of IL-6, IL-1β, and TNF were measured in vehicle-treated RAW264.7 cells as 26.12 ± 2.80 pg/mL, 28.70 ± 10.94 pg/mL, and 275.80 ± 61.51 ng/mL respectively. In contrast, in the LPS-treated group, the IL-6, IL-1β, and TNF levels increased to 4794.0 ± 216.40 pg/mL, 330.10 ± 3.83 pg/mL, and 10.62 ± 0.34 ng/mL, respectively. PAA treatment significantly prevented increases in IL-6, IL-1β, and TNF, compared with the LPS-only treated group, in a dose-dependent manner (Figure 4). The inhibitory effect of pestalotic acid A on pro-inflammatory cytokines production parallels its effect on NO production.

### 3.5. Pestalotic Acid A Blocks Phosphorylation of p65 NF-κB in LPS-Stimulated RAW264.7 Cells

**Figure 5:**
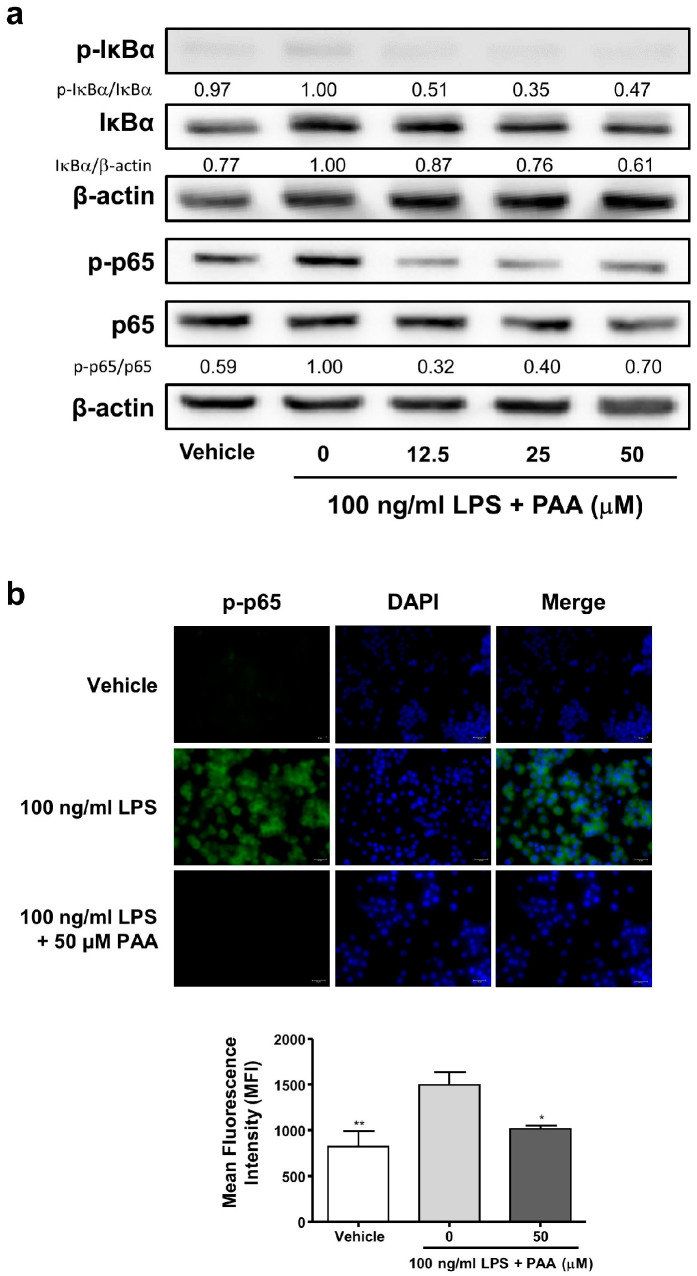
Pestalotic acid A inhibits phosphorylation of p65 in LPS-stimulated RAW264.7 cells. (**a**) Whole-cell lysates (10 μg) were subjected to 10% SDS-PAGE to analyze the NF-κB pathway (p-p65 and p-Iκ-Bα). β-actin expression served as a loading control. Western blot analysis was performed as described in the Materials and Methods section (*n* = 1). (**b**) p-p65 was visualized by immunofluorescence. The nuclei were counterstained with DAPI (blue). The stained cells were visualized with a fluorescence microscope at 100× magnification (*n* = 5). Statistical significance was determined based on differences when compared with 0 μM PAA (*p < 0.05, ** *p* < 0.01).

Given that NF-κB is a key transcription factor involved in the release of inflammatory mediators [14,15], we examined the phosphorylation of the p65 subunit of NF-κB (p-p65) to elucidate the molecular mechanisms underlying the anti-inflammatory effects of PAA. Western blot analysis was performed to determine the protein expression of p-p65 in whole-cell lysate from LPS-stimulated RAW264.7 cells. As illustrated in Figure 5a, when the intensity of p-p65/p65 in 0 μM PAA was 1.00, the protein expression of p-p65 NF-κB decreased in the cell lysates after treatment with pestalotic acid A for 24 h t 0.32 in 12.5 μM PAA. In addition, PAA also decreased the expression of p-IκBα after 4 h in the cell lysates (p-IκBα/IκBα intensity 1.00; 50 μM, 0.47). Fluorescence microscopy further demonstrated that PAA inhibited p65 phosphorylation (0 μM PAA MFI, 1494.0 ± 143.7; 50 μM PAA MFI, 1018.0 ± 33.6) (Figure 5b). These results suggest that PAA suppresses NF-κB p65 subunit activation.

**Figure 6:**
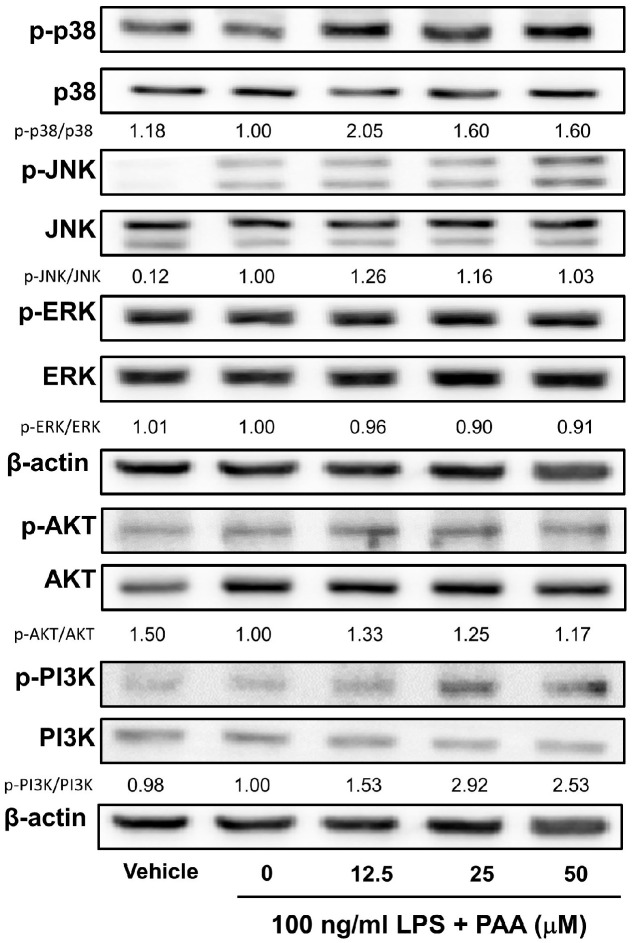
Pestalotic acid A does not inhibit the MAPK signaling pathway in LPS-stimulated RAW264.7 cells. Whole-cell lysates (10 μg) were subjected to 10% SDS-PAGE for the MAPK pathway (p-P38, p-JNK, and p-Erk). β-actin expression served as a loading control. Western blot analysis was performed as described in Materials and Methods (*n* = 1). Statistical significance was assessed relative to the 0 μM PAA.

To further elucidate the molecular mechanism by which p65 activation is inhibited, the effects of PAA on upstream signaling pathways regulating NF-κB activation, including the mitogen-activated protein kinase (MAPK) and phosphatidylinositol 3-kinase (PI3K)-AKT pathways, were assessed through Western blot analysis (Figure 6). It was found that PAA had no inhibitory effect on the MAPK pathways, but did induce phosphorylation of PI3K (p-PI3K/PI3K intensity 0 μM PAA; 1.00, 50 μM; 2.53). These data indicate that PAA exerts its anti-inflammatory effects primarily by inhibiting IκBα and NF-κB signaling pathways.

## 4. Discussion

PAA features a unique furylidene tetronic acid core, a structure that is rare among natural products [10]. Pestalotic acid A-I demonstrates strong antimicrobial activity against various plant pathogens, with minimum inhibitory concentrations ranging from 0.78 to 100 mg/mL [11]. This study is the first to elucidate the anti-inflammatory effects of PAA, a secondary metabolite isolated and identified from *P. vismiae*, an endophyte of Japanese holly, in LPS-stimulated macrophages.

Macrophages play a pivotal role in inflammation, acting as key regulators in both the initiation and resolution of inflammatory responses. They exhibit remarkable plasticity, allowing them to adapt their functions based on environmental cues, which is crucial for maintaining tissue homeostasis [16]. The role of macrophages in LPS-stimulated inflammation models is multifaceted, primarily involving the initiation, maintenance, and resolution of inflammatory responses. Macrophages respond to LPS by adopting pro-inflammatory phenotypes, releasing cytokines, and influencing other immune cells. This response is crucial for managing tissue inflammation and preventing excessive damage. There is currently a growing interest in discovering and developing new anti-inflammatory agents derived from natural products.

An increase in macrophage numbers is crucial for enhancing immune responses, as these cells are pivotal in both innate and adaptive immunity. Macrophages are essential for pathogen recognition, phagocytosis, and the secretion of pro-inflammatory cytokines, collectively bolstering the immune defense against infections and tumors [17]. PAA has been shown to induce macrophage proliferation, suggesting its potential to enhance immune response.

iNOS is an enzyme activated by inflammation that can produce large quantities of NO, which can be detrimental in excess. Increased NO levels from iNOS contribute to the inflammatory responses associated with conditions such as asthma and Barrett’s carcinogenesis [18,19]. The findings of this study indicated that PAA substantially reduced NO production in LPS-stimulated macrophages. This reduction in NO was attributed to the decreased iNOS expression at the protein level.

IL-6 is a pro-inflammatory cytokine that plays a role in the development of several autoimmune and chronic inflammatory conditions, including arthritis, peritonitis, and colitis [20,21]. Many autoimmune disorders are characterized by excessive IL-6 production. Tocilizumab, a humanized monoclonal antibody targeting the IL-6 receptor, inhibits IL-6-driven signaling and is approved for treating rheumatoid arthritis and Castleman’s disease. Furthermore, IL-6 levels are elevated in various cancers, and the amount of circulating IL-6 correlates with the prognosis in patients with cancer [20].

IL-1β is pivotal in macrophage function, particularly concerning inflammation and immune defense mechanisms. This cytokine is predominantly released by activated macrophages and is integral to managing inflammatory responses. The synthesis of IL-1β involves two key signals: initial pathogens recognition, which primes the macrophages, followed by inflammasome activation that processes pro-IL-1β into its active form [22]. As a major pro-inflammatory cytokine, IL-1β is essential in orchestrating the immune system’s response to pathogens. However, if its regulation is disrupted, it can lead to persistent inflammation and subsequent tissue damage [23].

Initially, TNF was identified owing to its tumor-fighting properties; however, it is now recognized as a critical mediator in inflammation. Activated by a broad spectrum of pathogenic stimuli, TNF stimulated the production of additional inflammatory mediators and proteases that manage inflammatory processes [24]. Moreover, TNF has been found to decrease sensitivity to corticosteroids [25]. Our studies have demonstrated that PAA effectively inhibits the production of IL-6, IL-1β, and TNF in LPS-stimulated murine macrophages.

The regulation of inflammatory mediators like NO, IL-6, IL-1β, and TNF is controlled by the crucial transcription factor, NF-κB [26]. Upon activation by inflammatory signals, NF-κB is released from its inhibitory protein, IκB-a. Subsequently, NF-κB translocates from the cytosol to the nucleus, where it binds to DNA, leading to the expression of various inflammatory mediators, including iNOS, as well as cytokines (IL-6, IL-1β, and TNF) [27]. Consequently, the development of anti-inflammatory agents has focused on downregulating the transcription of such pro-inflammatory mediators. Therefore, targeting NF-κB is a strategy for treating inflammation. As illustrated in Figure 5, pestalotic acid A inhibited the phosphorylation of the p65 NF-κB sub-unit. These findings demonstrate that pestalotic acid A suppressed the LPS-induced expression of iNOS and inflammatory cytokines (IL-6, IL-1β, and TNF).

The activation of NF-κB signaling in LPS-stimulated RAW264.7 cells involves multiple pathways. LPS, a component of the outer membrane of Gram-negative bacteria, is a potent activator of immune responses in macrophages, including RAW264.7 cells. The primary pathway for LPS-mediated NF-κB activation involves Toll-like receptor 4, which initiates the signaling cascade leading to NF-κB activation [28,29,30]. Furthermore, other pathways, including MAPKs and PI3K/Akt, also play roles in modulating NF-κB signaling in these cells. The MAPK pathway, including Erk, JNK, and p38, is activated in response to LPS. JNK and ERK, in particular, are involved in the inflammatory response and can influence NF-κB activity [31]. The PI3K/Akt pathway is another signaling route contributing to NF-κB activation. LPS stimulation leads to Akt phosphorylation, which is associated with the translocation of NF-κB to the nucleus [32]. In this study, it was found that PAA specifically inhibits the IκBα/NF-κB pathway, but does not inhibit the MAPK and PI3k pathways. PI3K pathways are essential for macrophage survival, being crucial for the induction of survival signals [33]. In this study, PAA induced activation of PI3K and increased the proliferation of macrophages. These findings mean that PAA induces the proliferation of macrophages by activating PI3K.

## 5. Conclusions

**Figure 7:**
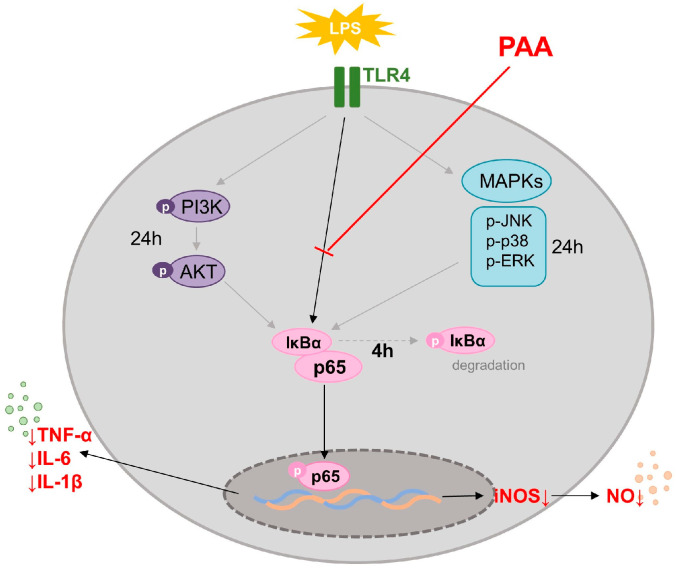
Anti-inflammatory effect of Pestalotic acid A.

This study is the first to elucidate the anti-inflammatory effects of PAA, a known polyketide-type secondary metabolite isolated from *Pestalotiopsis vismiae*, PAA significantly reduces the production of inflammation-related mediators, including NO, IL-6, IL-1β, and TNF. PAA inhibits expression of iNOS by suppressing the activation of NF-κB pathway (Figure 7). These findings suggest that PAA is a promising candidate for the development of therapeutic agents for inflammatory diseases.

## Data Availability

The data presented in this study are available on request from the corresponding author.

## Supplementary Materials

The following supporting information can be downloaded at: https://www.mdpi.com/article/10.3390/biomedicines13061445/s1.

## Abbreviations

DMEM	Dulbecco’s modified Eagle’s medium
DMSO	dimethyl sulfoxide
EDTA	ethylenediamine tetraacetic acid
EtOAc	ethyl acetate
ESI-MS	electrospray ionization mass spectrometry
FBS	fetal bovine serum
HMBC	heteronuclear multiple-bond correlation spectroscopy
HPLC	high-performance liquid chromatography
iNOS	inducible nitric oxide synthase
IL-1β	interleukin-1 beta
IL-6	interleukin-6
LPS	lipopolysaccharide
MAPK	mitogen-activated protein kinase
MPLC	medium-pressure liquid chromatography
NMR	nuclear magnetic resonance
NO	nitric oxide
PDA	potato dextrose agar
TNF-α	tumor necrosis factor-alpha
