# Supplementary material for: Anti-Inflammatory Effect of Pestalotic Acid A Derived from Pestalotiopsis vismiae, an Endophytic Fungus of Ilex prenatal, in Lipopolysaccharide-Stimulated RAW264.7 Cells
Source: Biomedicines. 2025 Jun 12;13(6):1445. doi: 10.3390/biomedicines13061445 (PMC12191257; doi:10.3390/biomedicines13061445)
Supplement: Supplementary file 1 [file biomedicines-13-01445-s001.zip › biomedicines-3650236-supplementary.pdf]

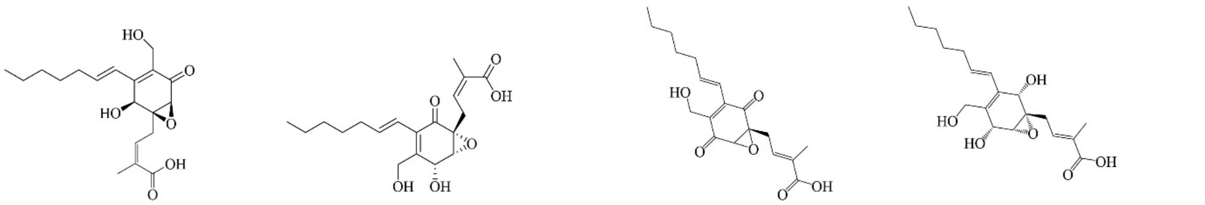

Pestalotic acid A

Ambuic acid

Pestalic acid E

(2E)-4-[(1S,2S,5R,6R)-3-(1E)-1-Hepten-1-yl-2,5-dihydroxy-4-(hydroxymethyl)-7-oxabicyclo[4.1.0]hept-3-en-1-yl]-2-methyl-2-butenoic acid

Figure S1. The structure of four polyketide-types secondary metabolites isolated from *Pestalotiopsis vismiae*

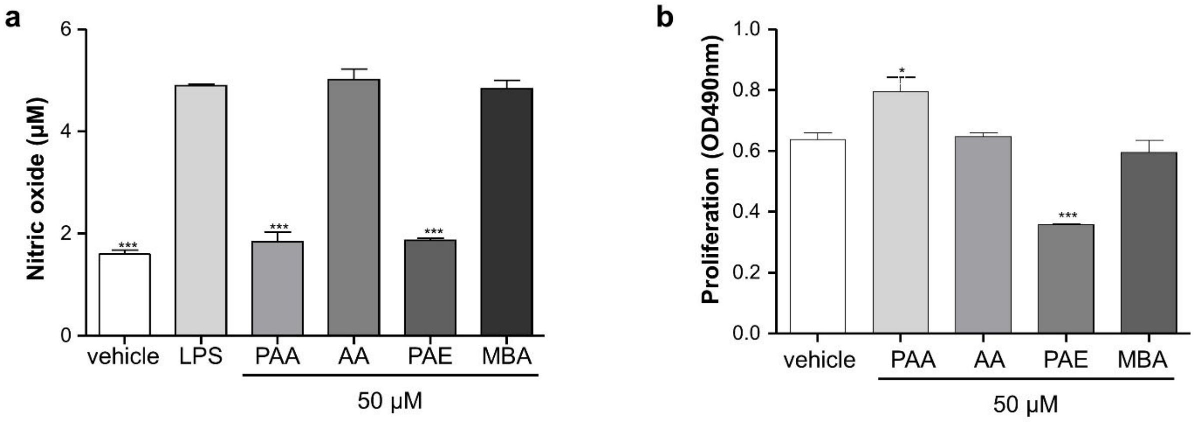

Figure S2. The effect of four polyketide-types secondary metabolites on nitric oxide (a) and cell proliferation (b) (n=3). Statistical significance was determined based on differences when compared with LPS (a) or vehicle (b) ($^{*}P < 0.05$, $^{**}P < 0.01$, $^{***}P < 0.001$). Abbreviations: PAA: Pestalotic acid A, AA: Ambuic acid, PAE: Pestalic acid E, MBA: (2E)-4-[(1S,2S,5R,6R)-3-(1E)-1-Hepten-1-yl-2,5-dihydroxy-4-(hydroxymethyl)-7-oxabicyclo [4.1.0]hept-3-en-1-yl]-2-methyl-2-butenoic acid

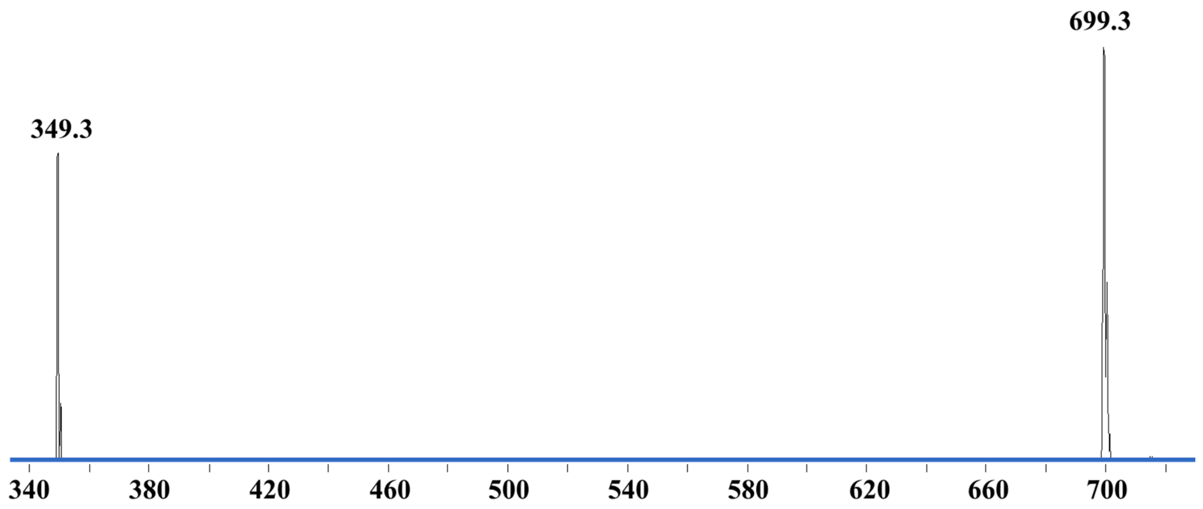

Figure S3 ESI-MS spectrum of compound **1**.

Table S1. ^{1}H(500MHz) and ^{13}C (125MHz) NMR spectroscopic data for pestalotic acid A (**1**) (δ_H, mult., *J* in Hz).

No.	Pestalotic acid A (**1**)	
	δ_C	δ_H
1	171.2	
2	132.8	
3	135.9	6.83 (td, 7.5, 1.3)
4	30.9	3.15 (dd, 15.8, 7.6)<br>2.72 (dd, 15.8, 7.6)
5	64.5	
6	66.3	4.85 (s)
7	151.2	
8	130.2	
9	197.0	
10	59.0	3.35 (s)
11	127.1	6.66 (dt, 15.9, 1.2)
12	143.1	6.54 (dt, 15.9, 7.0)
13	35.0	2.29 (dd, 15.0, 8.0)
14	29.6	1.52 (m)
15	32.6	1.35 (m)*
16	23.7	1.35 (m)*
17	14.4	0.92 (t, 7.0)
18	12.9	1.89 (s)
19	55.0	4.48 (d, 11.7)<br>4.28 (d, 11.7)

* Overlapping resonances within the same column.

Abbreviation: NMR: nuclear magnetic resonance

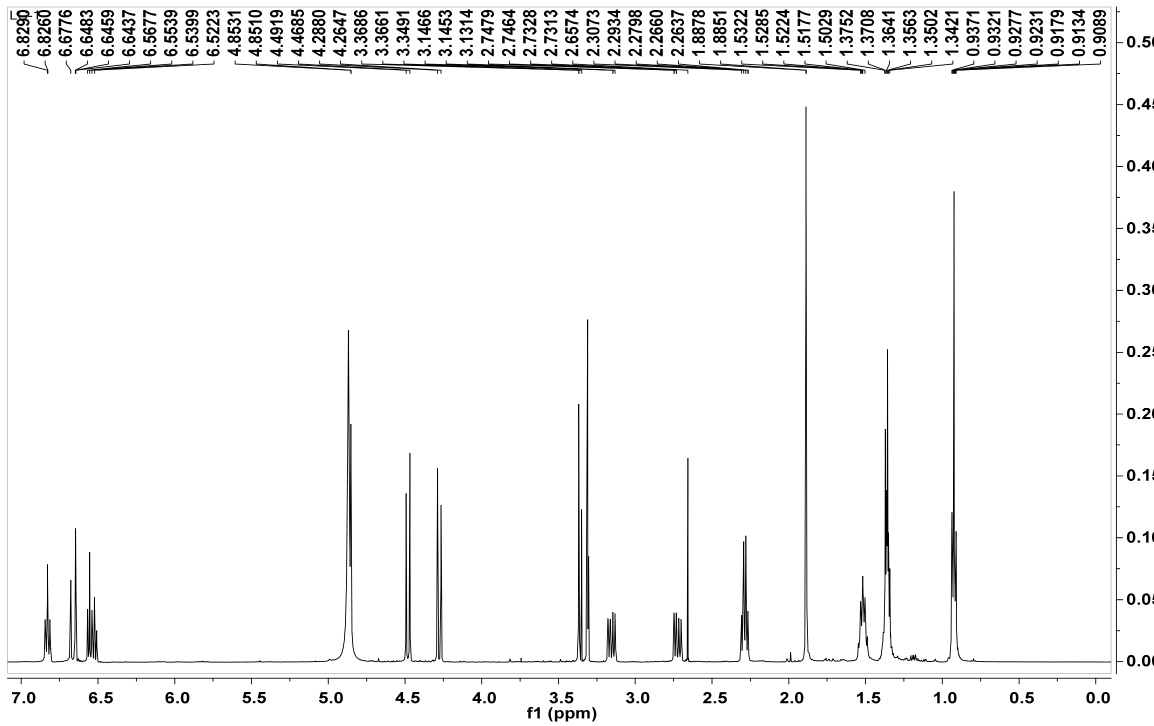

Figure S4 ^{1}H NMR spectrum of compound **1**.

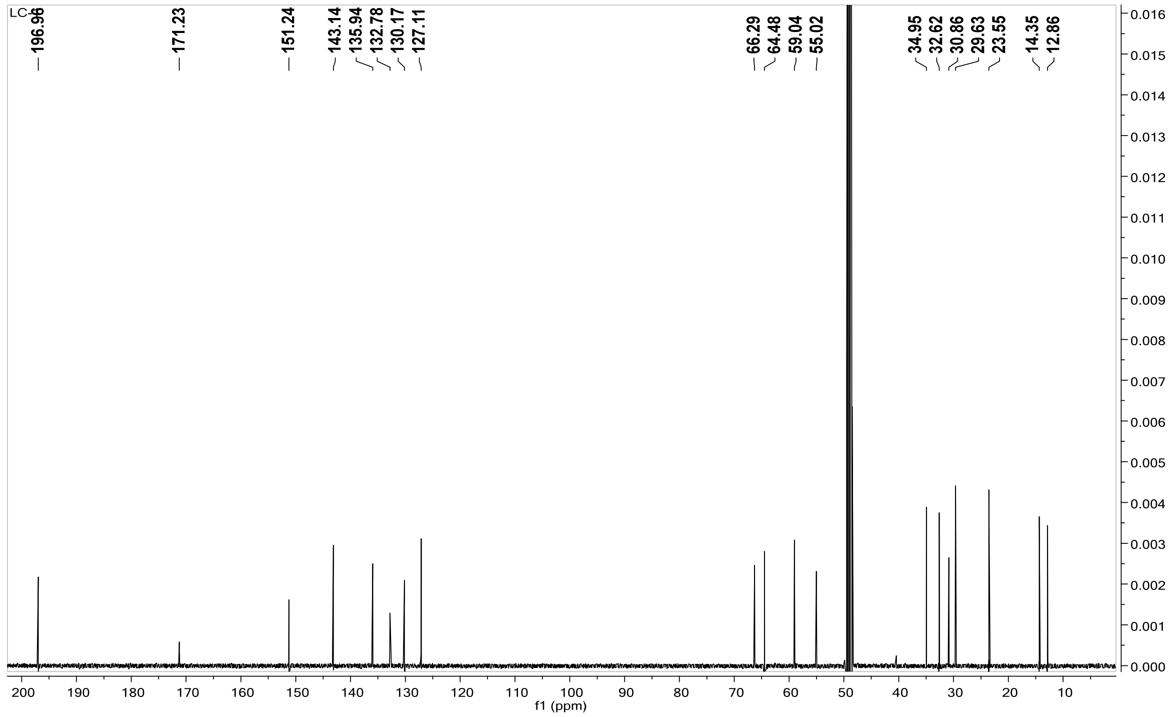

Figure S5 ^{13}C NMR spectrum of compound **1**.

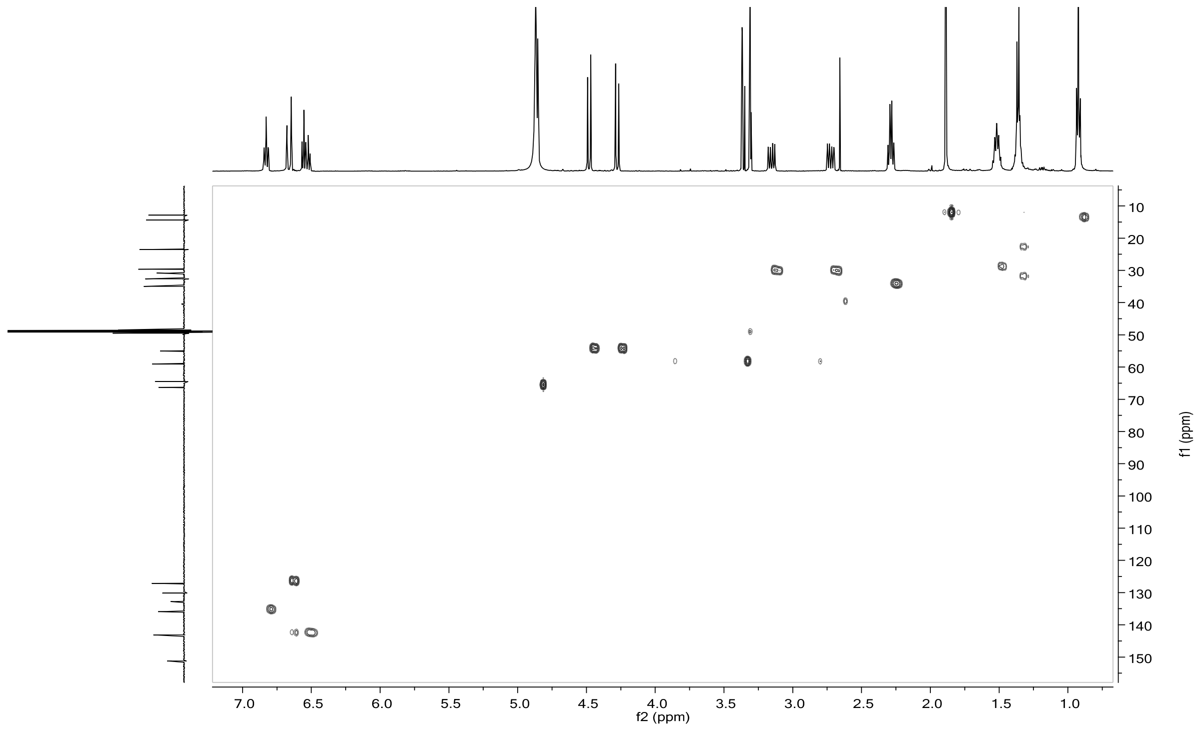

Figure S6. HMQC spectrum of compound **1**.

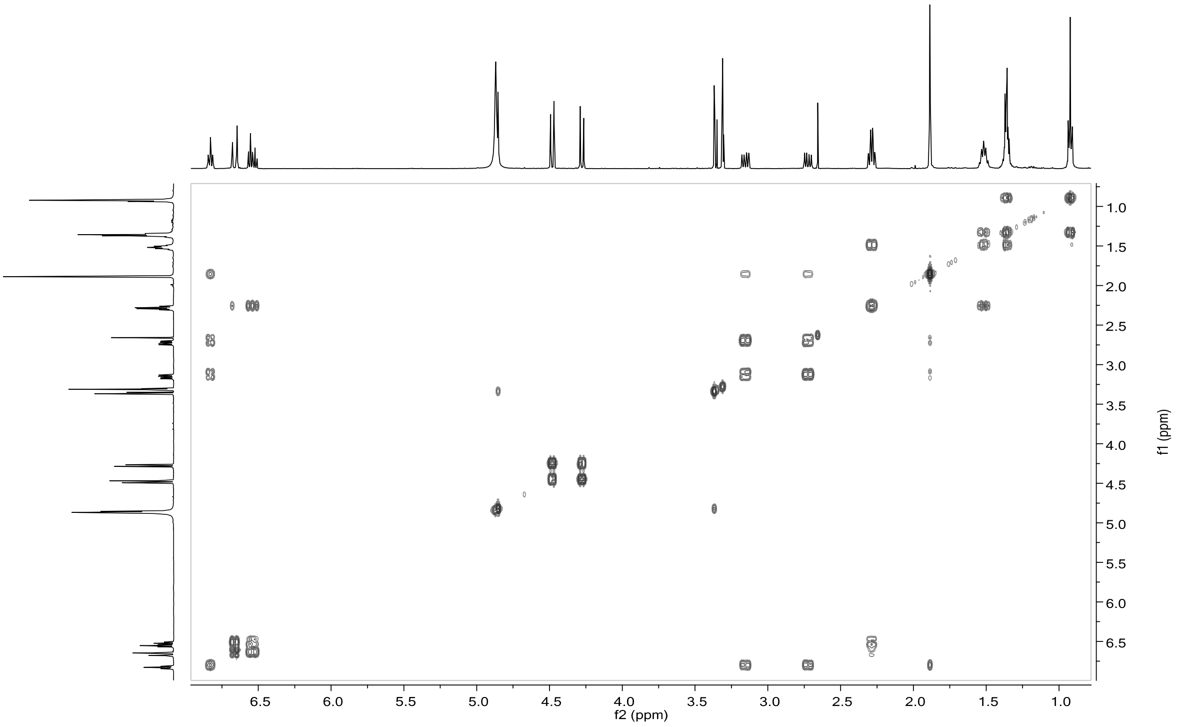

Figure S7. ^{1}H-^{1}H COSY spectrum of compound **1**.

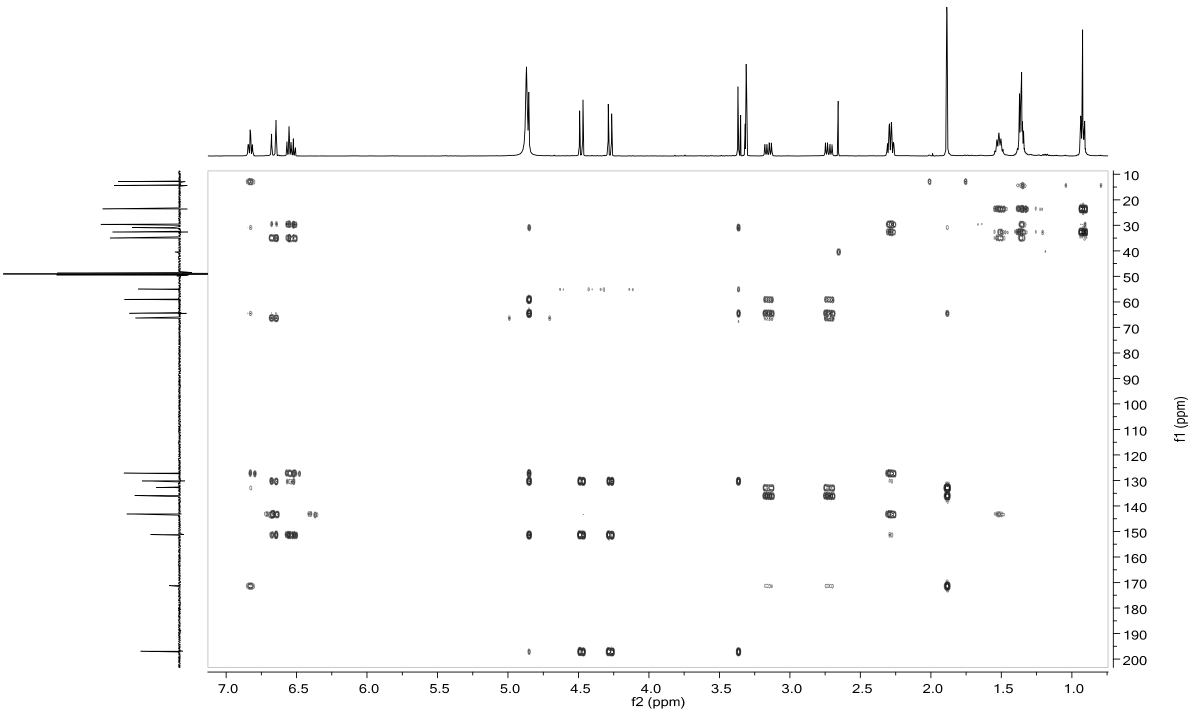

Figure S8. HMBC spectrum of compound **1**.

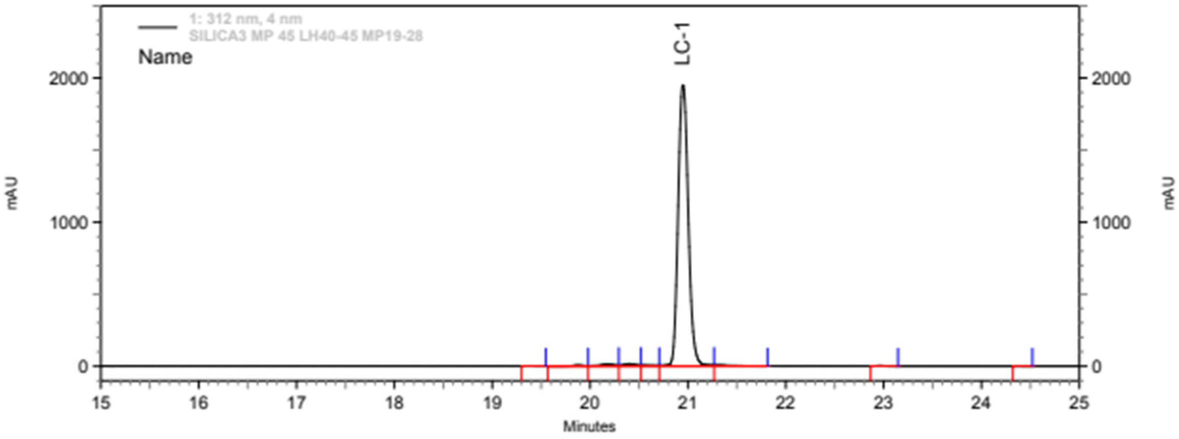

1: 312 nm, 4 nm Results

Name	Area Percent	Retention Time
LC-1	97.158	20.947

Figure S9. HPLC analysis of compound **1**.
